# Decrease in skeletal muscle index one year after radical cystectomy as a prognostic indicator in patients with urothelial bladder cancer

**DOI:** 10.1590/S1677-5538.IBJU.2018.0530

**Published:** 2019-09-02

**Authors:** Yun-Sok Ha, Sang Won Kim, Tae Gyun Kwon, Sung Kwang Chung, Eun Sang Yoo

**Affiliations:** 1 Department of Urology, School of Medicine, Kyungpook National University, Daegu, Republic of Korea

**Keywords:** Urinary Bladder Neoplasms, Sarcopenia, Survival

## Abstract

**Purpose:**

The present study aimed to determine whether sarcopenia after radical cystectomy (RC) could predict overall survival (OS) in patients with urothelial bladder cancer (UBC).

**Materials and Methods:**

The lumbar skeletal muscle index (SMI) of 80 patients was measured before and 1 year after RC. The prognostic significance of sarcopenia and SMI decrease after RC were evaluated using Kaplan–Meier analysis and a multivariable Cox regression model.

**Results:**

Of 80 patients, 26 (32.5%) experienced sarcopenia before RC, whereas 40 (50.0%) experienced sarcopenia after RC. The median SMI change was -2.2 cm2/m2. Patients with sarcopenia after RC had a higher pathological T stage and tumor grade than patients without sarcopenia. Furthermore, the overall mortality rate was significantly higher in patients with sarcopenia than in those without sarcopenia 1 year after RC. The median follow-up time was 46.2 months, during which 22 patients died. Kaplan-Meier estimates showed a significant difference in OS rates based on sarcopenia (P=0.012) and SMI decrease (P=0.025). Multivariable Cox regression analysis showed that SMI decrease (≥2.2 cm2/m2) was an independent predictor of OS (hazard ratio: 2.68, confidence interval: 1.007-7.719, P = 0.048).

**Conclusions:**

The decrease in SMI after surgery might be a negative prognostic factor for OS in patients who underwent RC to treat UBC.

## INTRODUCTION

Bladder cancer (BC) is one of the most common urinary tract malignancies worldwide (1-3). It is generally treated using transurethral resection of bladder tumor (TUR-BT) or radical cystectomy (RC), and systemic cisplastin-based chemotherapy is performed in cases of advanced or metastatic BC. However, because BC is a highly malignant tumor with a variable and unpredictable biologic potential, the survival forecast for patients remains poor (4, 5) The prognosis of BC is poor in elderly people and in those with serious comorbidities and poor performance status (6).

RC is the customary treatment for patients with muscle-invasive BC (MIBC), and it is also commonly used to treat selected patients with high-risk, non-muscle-invasive BC (NMIBC). A recent study reported that complications following RC are strongly associated with patient-related factors, such as age, performance status, and comorbidities (7). Moreover, numerous studies have demonstrated that frailty is associated with impaired mobility, disability, poor endurance, and prolonged hospitalization (8, 9). In particular, sarcopenia—skeletal muscle wasting— is a crucial physiological alteration underlying frailty that can emerge as a result of aging and malignant disease (10), and it has been identified as a prognostic factor for various cancers (11). In patients with BC who have undergone RC, sarcopenia is associated with poor survival (12). However, changes in the skeletal muscle index (SMI) after RC have not been established as a prognostic tool. The aim of the present study was to evaluate changes in the SMI 1 year after RC as a predictor of overall survival (OS) in patients with urothelial bladder cancer (UBC).

## MATERIALS AND METHODS

### Ethics statement

The ethics committee of Kyungpook National University Hospital reviewed and approved the current study protocol (approval number: KNUMC 2016-05-021). The study was performed in accordance with the ethical standards laid down in the 1964 Declaration of Helsinki and its later amendments. This was a retrospective study performed after approval from the institutional review board, who stated that consent was not required.

### Patients

The present study included 80 patients with non-metastatic UBC who had undergone RC (31 robot-assisted RCs and 49 open RCs) between August 2008 and May 2013 and who had serial axial computed tomography (CT) images showing sarcopenia both before and 1 year after the RC. Before RC, all patients underwent TUR-BT. Following histopathological examination and imaging studies, RC was performed. The indications for RC were as follows: MIBC without evidence of distant metastasis (clinical stage: T2–T4, Nx, M0), recurrent multifocal NMIBC refractory to repeated transurethral resection, and Bacille Calmette-Guerin (BCG)-resistant carcinoma *in situ*. The exclusion criteria were as follows: previous pelvic radiation, clinical stage M1, and prior combination surgery. Open RC was performed through a midline incision in the typical manner (13). Robot-assisted RC was performed using the same surgical procedure as reported by Bak et al. (1). Standard pelvic lymphadenectomy (both obturator and external iliac nodes) was performed in all patients, except for 1 patient undergoing robot-assisted RC and 6 undergoing open RC because of severe adhesions. The clinical T stage was based on the guidelines of the 2010 American Joint Committee on Cancer TNM staging system for BC (14). Histological grades were determined according to the 2004 World Health Organization (WHO) classification system (15). Patients with cT3, cT4, and node-positive disease (based on the analysis of CT images), but with good performance status, received at least 3 cycles of cisplatin-based neoadjuvant chemotherapy. Each patient was followed up and managed according to standard practice (16).

### Image analysis

Patients underwent abdominal CT for initial cancer staging and routine diagnostic purposes. For each patient, a set of CT scans just before and a mean of 1 year after RC was selected. Quantitative assessment of muscle areas was performed using commercially available software (Terarecon 4.4.7, San Mateo, CA, USA) by a subspecialty-trained urogenital radiologist. The radiologist selected the single cross-sectional areas at the level of the third lumbar vertebrae (L3) in which both transverse processes could be fully seen. The cross-sectional areas (cm^2^) of all skeletal muscles at L3 were computed automatically by summing the appropriate pixels within the CT Hounsfield unit (HU) range of -29 HU to 150 HU (17). After applying a predefined HU threshold set for each slice, boundaries between the different tissues were corrected manually when necessary.

### Definition of sarcopenia

**Figure 1:**
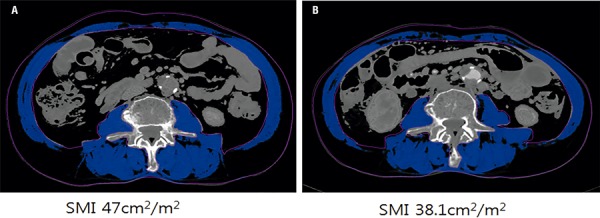
Representative CT scans with decreased skeletal muscle index (SMI) in an 82-year-old man before (A) and at a mean of one year (B) after radical cystectomy.

Muscle area was normalized for the square of patient height in meters (m^2^) and reported as the lumbar SMI index (cm^2^/m^2^) (18, 19). In Figure-1 shows the CT scans and SMI values of an 82-year-old man before and at a mean of 1 year after RC. Sarcopenia was defined as a lumbar SMI of <43 cm^2^/m^2^ for men with a body mass index (BMI) of <25 kg/m^2^, as a lumbar SMI of <53 cm^2^/m^2^ for those with a BMI of ≥25 kg/m^2^, and as an SMI of <41 cm^2^/m^2^ for women, as recommended by Martin et al. (20).

### Statistical analysis

Patients were divided into two groups of 40 based on their sarcopenic status one year after RC: non-sarcopenic patients and sarcopenic patients. Differences between the groups were evaluated using the chi-square test for categorical variables and Student’s t-test for continuous variables. Multivariable Cox proportional hazards models were used to test the associations between the variables and OS, with hazard ratios (HRs) and 95% confidence intervals (CIs) calculated for each factor. OS was measured from the date of diagnosis to death or final follow-up. All statistical analyses were performed using the Statistical Package for the Social Sciences, version 18.0 (SPSS Inc., Chicago, IL, USA), and *P-values* < 0.05 were considered statistically significant.

## RESULTS

Of the 80 patients, 26 (32.5%) were sarcopenic before RC, whereas 40 (50.0%) were sarcopenic after RC. The median change in SMI was -2.2 cm^2^/m^2^. In Table-1 presents patient demographics and preoperative characteristics according to SMI 1 year after RC. Age, sex, and BMI were not significantly associated with sarcopenia 1 year after RC (*P*>0.05). The mean preoperative SMI was 50.51 cm^2^/m^2^ in non-sarcopenic patients, significantly higher than that in sarcopenic patients (43.76 cm^2^/m^2^; *P*<0.001). In the cohort of patients with sarcopenia after RC, 47.5% had been classified as sarcopenic preoperatively, while only 7 (17.5%) patients with preoperative sarcopenia were in the non-sarcopenic group after surgery (*P*=0.004). Higher clinical stage (≥T2) at latest TUR-BT was more prevalent in sarcopenic patients than in those without sarcopenia (65.0% *vs*. 37.5%; *P*=0.014). Sarcopenia was not significantly associated with ASA classification, presence of carcinoma *in situ*, BCG instillation history, and neoadjuvant chemotherapy.

**Table 1:** Patient demographics and preoperative characteristics according to skeletal muscle index one year after radical cystectomy.

Parameters	Non-sarcopenic patients, (*n* = 40)	Sarcopenic patients, (*n* = 40)	*P*-value
**Age, years**			**0.262**
<70	24 (60.0)	19 (47.5)	
≥70	16 (40.0)	21 (52.5)	
**Sex**			**0.363**
Male	35 (87.5)	32 (80.0)	
Female	5 (12.5)	8 (20.0)	
BMI (kg/m^2^)	23.41 ± 3.41	22.39 ± 2.71	0.142
Preoperative SMI (cm^2^/m^2^)	50.51 ± 8.41	43.76 ± 6.40	< 0.001
**Preoperative sarcopenia**			**0.004**
No	33 (82.5)	21 (52.5)	
Yes	7 (17.5)	19 (47.5)	
**ASA classification**			**0.499**
1	6 (15.0)	4 (10.0)	
≥2	34 (85.0)	36 (90.0)	
**Clinical stage at latest TUR-BT**			**0.014**
≤T1	25 (62.5)	14 (35.0)	
≥T2	15 (37.5)	26 (65.0)	
**Presence of CIS at latest TUR-BT**			**0.712**
No	35 (87.5)	37 (92.5)	
Yes	5 (12.5)	3 (7.5)	
**BCG instillation history**			**0.712**
No	37 (92.5)	35 (87.5)	
Yes	3 (7.5)	5 (12.5)	
**Neoadjuvant chemotherapy**			**0.762**
No	34 (85.0)	33 (82.5)	
Yes	6 (15.0)	7 (17.5)	

**ASA =** American Society of Anesthesiologists; **BCG =** Bacille Calmette-Guerin; **BMI =** body mass index; **CIS =** Carcinoma in situ; **SMI =** Skeletal muscle index; **TUR-BT =** Transurethral tumor resection of bladder tumor

In Table-2 shows the relationship between sarcopenia after RC and clinicopathological features. Evaluation revealed that patients with sarcopenia had higher tumor stage and grade than those without sarcopenia 1 year after RC. The mean changes in SMI were -3.80 cm^2^/m^2^ in sarcopenic patients and -1.12 cm^2^/m^2^ in non-sarcopenic patients (*P*=0.001). Notably, patients with sarcopenia had significantly higher all-cause mortality rates than those without sarcopenia (*P*=0.012). Metastasis rate did not differ significantly between the 2 groups (*P*=0.104).

**Table 2:** Comparison of clinicopathological variables according to skeletal muscle index one year after radical cystectomy.

Parameters	Non-sarcopenic patients, (n=40)	Sarcopenic patients, (n=40)	P-value
**Pathological stage**			**0.028**
T0, Tis, Ta	6 (15.0)	2 (5.0)	
T1	15 (37.5)	10 (25.0)	
T2	10 (25.0)	9 (22.5)	
T3	4 (10.0)	13 (32.5)	
T4	5 (12.5)	6 (15.0)	
**Histological grade**			**0.029**
Low	8 (20.0)	1 (2.5)	
High	32 (80.0)	39 (97.5)	
**Lymph node involvement**			**0.617**
No	28 (70.0)	30 (75.0)	
Yes	12 (30.0)	10 (25.0)	
**Lymphovascular invasion**			**0.712**
No	37 (92.5)	35 (87.5)	
Yes	3 (7.5)	5 (12.5)	
SMI changes 1 year after RC (cm^2^/m^2^)	-1.12±3.14	-3.80±3.59	0.001
Median follow-up period (months, range)	48.1 (14.4-105.1)	43.5 (12.5-93.0)	0.060
**Metastasis**			**0.104**
No	29 (72.5)	22 (55.0)	
Yes	11 (27.5)	18 (45.0)	
**Overall death**			**0.012**
No	34 (85.0)	24 (60.0)	
Yes	6 (15.0)	16 (40.0)	

**Figure 2:**
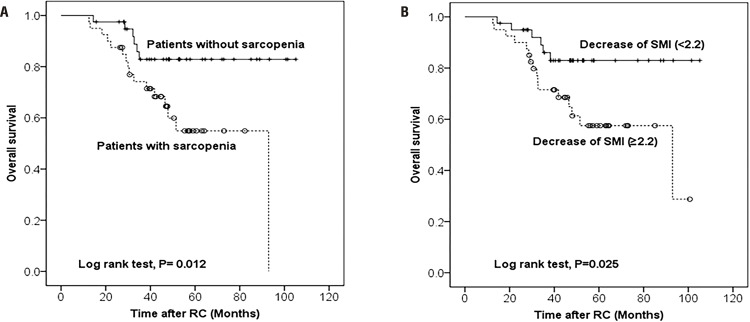
Kaplan-Meier curve depicting overall survival according to postoperative sarcopenia (A) and change in skeletal muscle index (SMI) (B).

Twenty-two patients died during the median follow-up of 46.2 months. Kaplan-Meier estimates showed a significant difference in OS (*P*=0.012) and SMI decrease (*P*=0.025) between the 2 groups (Figure-2). In Table-3, we assessed the relationship between various measured parameters and OS. Although sarcopenia 1 year after RC was significantly associated with OS in univariable analysis, there was no statistical association between sarcopenia itself and OS in multivariable Cox analysis. As indicated by the multivariable analysis, the probability of OS increased greatly as SMI decreased. When other factors were adjusted for, pathological T stage and an SMI decrease of ≥2.2 cm^2^/m^2^ (HR: 2.689, 95% CI: 1.007-7.719, *P*=0.048) were found to be independent predictors of OS.

**Table 3:** Multivariable Cox regression analysis of factors predicting overall death in patients with bladder cancer after radical cystectomy.

Parameters	HR	95% CI		P-value	HR	95% CI		P-value
Age	1.002	0.955	1.050	0.943	1.017	0.974	1.063	0.434
Gender	0.359	0.078	1.660	0.190	0.615	0.137	2.768	0.527
Pathological T stage	1.835	1.191	2.827	0.006	1.664	1.062	2.607	0.026
Lymph node involvement	1.329	0.515	3.424	0.556	1.719	0.675	4.383	0.256
Grade	1.315	0.133	12.977	0.815	1.381	0.149	12.815	0.776
Sarcopenia 1 year after RC (No vs. Yes)	1.714	0.645	4.558	0.280				
SMI decrease (< 2.2 vs. ≥ 2.2 cm^2^/m^2^)					2.689	1.007	7.719	0.048

**CI =** confidence interval; **HR =** hazard ratio; **SMI =** skeletal muscle index

## DISCUSSION

Muscle loss is expected in the elderly and is a rising concern in patients with cancer. Sarcopenia is characterized by decrease in protein synthesis and an increase in protein degradation (21). Hence, the condition displays many similar characteristics and can be a broad and integrated sign of cancer cachexia. Several recent studies have revealed definite connections between sarcopenia and mortality after RC to treat UBC (12, 22). To our knowledge, our current report was the first to indicate that changes in SMI after RC are associated with OS in patients with UBC. In particular, we observed greater all-cause mortality among patients who were sarcopenic after RC to treat UBC (40.0% v*s.* 15.0% in non-sarcopenic patients; *P*=0.012). The median OS was 43.5 months among patients with sarcopenia versus 48.1 months among those with normal SMI after RC. All-cause mortality was more prevalent in the sarcopenia group than in the cohort without sarcopenia, according to Kaplan-Meier analysis (log-rank test: *P*=0.012; Figure-2A). Likewise, Kaplan-Meier analysis also revealed that patients with larger SMI changes (≥2.2 cm^2^/m^2^) had a worse OS rate than those with smaller changes (<2.2 cm^2^/m^2^; log rank test; *P*=0.0025; Figure-2B). In our multivariable analysis, larger SMI decreases (≥2.2 cm^2^/m^2^) were associated with the risk of all-cause mortality (HR: 2.689, 95% CI: 1.007-7.719, *P*=0.048). Taken together with our results, sarcopenia and decreased SMI after RC are clinically useful and highly objective predictors of OS in patients with UBC who have undergone RC. Although sarcopenia one year after RC was significantly associated with OS in univariable analysis, there was no statistical association between sarcopenia itself and OS in multivariable Cox analysis. This suggested that the change in SMI was more useful for prediction of the OS after RC.

In this regard, previous studies have also reported that lower SMI and sarcopenia are modifiable prognostic factors in patients with UBC who have undergone RC. For instance, Psutka et al. showed that preoperative sarcopenia was independently associated with both increased cancer-related death and all-cause mortality in a multivariable analysis (22). In another study by Hirasawa et al. involving patients with UBC, preoperative sarcopenia was a significant independent predictor of unfavorable outcome, clinical T stage, hydronephrosis, histological type of TUR-BT specimens, and neutrophil-to-lymphocyte ratio (12). Conversely, a report by Smith et al. implied that sarcopenia was not significantly associated with worse OS rate (23). In the present study, preoperative sarcopenia was not associated with any clinicopathological features or prognoses. However, sarcopenia one year after RC was significantly associated with various pathological features, including tumor state (*P*=0.028), tumor grade (*P*=0.029), and OS. We also observed that SMI change was a useful predictor of OS after RC. Therefore, we suggest that postoperative CT should be performed and that clinicians should check the SMI during follow-up in patients with UBC. Nutritional support and the prevention of cachexia might be needed in selected patients with UBC who have undergone RC.

Sarcopenia may also predict complications and OS among patients with advanced or metastatic UBC who have undergone RC (21, 23, 24). In this regard, Wan et al. revealed that low SMI was frequently found in patients with BC who had undergone RC, and that this was strongly associated with early complications after surgery (24). Similarly, Smith et al. reported that sarcopenia was a predictor of major complications after RC in women, even after adjustment for known risk stratification characteristics (23). In advanced UBC, sarcopenia was useful in evaluating prognosis (21). More specifically, in a cohort of 88 patients with advanced UBC, the median OS rates were 11 and 31 months among sarcopenic and non-sarcopenic patients, respectively. In a multivariable analysis, sarcopenia was a significant and independent predictor of shorter OS (HR: 3.36). As sarcopenia reflects many clinical conditions, such as frailty, low nutritional status, active catabolism, and systemic inflammation, clinicians, including uro-oncologists, may use it for various purposes.

Several limitations of the current study must be acknowledged. First, the study had a retrospective design and involved a relatively small number of patients who underwent RC at a single institution. This may have led to sampling bias. Moreover, patients without available CT scans were excluded, which may also have caused selection bias. A prospective, randomized study involving a larger cohort and using multi-institutional methods will be required to confirm the present results. Second, the definition of sarcopenia was diverse in the present study. Although the volume of skeletal muscle mass differs according to ethnicity (25), the cut-off ranges defined in a previous Western study were within those determined in the present study. Considering ethnic and constitutional factors, a validated definition should be adopted in further studies. Despite these drawbacks, our present study presents a novel prognostic marker for predicting OS in patients with UBC who have undergone RC. The study indicated that the correction of sarcopenia after RC, as well as surveillance in selected patients, will improve postoperative management.

## CONCLUSIONS

Sarcopenia and SMI changes one year after RC, which can be readily followed up using routine CT, might be effective predictors of OS in patients with UBC. This novel prognostic marker may assist in selecting patients with UBC who would benefit from nutritional support and interventions to prevent muscle wasting and consequent sarcopenia. The clinical utility of SMI changes as a prognostic marker merits further evaluation in prospective or external validation studies.
